# Digitalized Design of Extraforaminal Lumbar Interbody Fusion: A Computer-Based Simulation and Cadaveric Study

**DOI:** 10.1371/journal.pone.0105646

**Published:** 2014-08-26

**Authors:** Mingjie Yang, Cheng Zeng, Song Guo, Jie Pan, Yingchao Han, Zeqing Li, Lijun Li, Jun Tan

**Affiliations:** Department of Spine Surgery, Shanghai East Hospital, Tongji University School of Medicine, Shanghai, People's Republic of China; University of Michigan, United States of America

## Abstract

**Purpose:**

This study aims to investigate the feasibility of a novel lumbar approach named extraforaminal lumbar interbody fusion (ELIF), a newly emerging minimally invasive technique for treating degenerative lumbar disorders, using a digitalized simulation and a cadaveric study.

**Methods:**

The ELIF surgical procedure was simulated using the Mimics surgical simulator and included dissection of the superior articular process, dilation of the vertebral foramen, and placement of pedicle screws and a cage. ELIF anatomical measures were documented using a digitalized technique and subsequently validated on fresh cadavers.

**Results:**

The use of the Mimics allowed for the vivid simulation of ELIF surgical procedures, while the cadaveric study proved the feasibility of this novel approach. ELIF had a relatively lateral access approach that was located 8–9 cm lateral to the median line with an access depth of approximately 9 cm through the intermuscular space. Dissection of the superior articular processes could fully expose the target intervertebral discs and facilitate a more inclined placement of the pedicle screws and cage with robust enhancement.

**Conclusions:**

According to the computer-based simulation and cadaveric study, it is feasible to perform ELIF. Further research including biomechanical study is needed to prove ELIF has a superior ability to preserve the posterior tension bands of the spinal column, with similar effects on spinal decompression, fixation, and fusion, and if it can enhance post-fusion spinal stability and expedites postoperative recovery.

## Introduction

Lumbar interbody fusion (LIF), such as posterior lumbar interbody fusion (PLIF) and transforaminal lumbar interbody fusion (TLIF), is the mainstay surgical treatment for degenerative lumbar disease, lumbar instability, and intervertebral disc disorders. Spinal surgeons are always attempting to modify the surgical approaches to LIF in more minimal invasive ways. These newly emerging modifications include anterior LIF, with a trans- or extraperitoneal approach anterior to the lumbar vertebrae [1], extreme lateral LIF, with a trans-psoas-major-muscle approach that is lateral to the lumbar vertebrae [2], and axial LIF, with a presacral approach [3]. However, these modified techniques are subject to some limitations including a steep learning curve, technical difficulty of manipulation, and high risk of procedural complications such as retrograde ejaculation, vascular or ureteral injury, and compromised lumbosacral plexus or genitofemoral nerve function [4], [5]. Therefore, conventional PLIF and TLIF are still preferred in current practice [6].

Inspired by the conception of transforaminal endoscopy (the Tessys technique), we developed a modified TLIF technique, namely extreme lateral TLIF (ELIF) [7]. ELIF has a more lateral access approach than TLIF, avoiding the inferior articular process and allowing for full exposure of the superior counterpart, which causes the nerve compression-associated symptoms. ELIF also facilitates the decompression of the lateral vertebral canal and the fusion of vertebral bodies in a “safety triangle.”

The primary objective of this study was to assess the feasibility of ELIF using a computer-based simulation and to validate the digitalized anatomic measurements using a cadaveric model.

## Materials and Methods

### Ethics Statement

This study has been reviewed and approved by ethics committee of Shanghai East Hospital, Tongji University school of medcine before the study began. And the ethic statement form has been upload in the attach files.

In this study we use DICOM data of a patient, and the patient's written informed consent was provided.

Our research also involved the use of two fresh lumbar cadaveric specimens which were provided by the Department of Anatomy, Shanghai Medical college, Fudan University. This department is also a donation centre with the URL:http://www.redcross-sha.org/view.aspx?id=5199. And the written informed consent from the donor were obtained for use of this sample in research.

### Three-dimensional modeling and simulated ELIF

A 26-year-old man (height, 172 cm; weight, 67 kg; body mass index, 22.6 kg/m^2^) diagnosed with an L_4/5_ lumbar disc herniation was recruited for a lumbar computed tomography (CT) scan preoperatively from the T_12_ level to the entrance of the pelvis using a dual source scanner (SOMATOM Definition Flash; Siemens Medical Solutions Inc., Forchheim, Germany). The scanning parameters were as follows: tube current  = 250 mA, tube voltage  = 120 kV, scanning slice thickness  = 1.0 mm, and reconstruction slice thickness  = 1.0 mm. Digital imaging and communications in medicine (DICOM) data obtained from the scanning were imported into the Mimics V14.0 system (Materialise NV, Leuven, Belgium) for image post-processing. The region of interest was selected and reconstructed using the automatic reconstruction module. The bone tissue threshold was also automatically set to clearly visualize the anatomy in the region of interest and produce the mask of the target tissue. The region growing technique was used to acquire the masks for the femoral head, pelvis, sacrum, and five lumbar vertebrae, each of which was independently processed and individually colored. The masks were selected for further three-dimensional (3D) reconstruction and smoothing to generate the 3D solid model for the bony pelvis, including the lumbar and sacral vertebrae.

The L_5_ vertebral body was separately extracted, and the superior articular processes were partially (approximately 3/4) dissected using the simulated cutter with an appropriate angle. The processed L_5_ vertebral body was restored to the original spinal column. The bases of the superior articular process residuals were shown to partially articulate with the inferior articular processes; however, the intervertebral foramen was dilated roughly 30% compared to prior to the operation. This enlarged foramen allowed the surgeon to perform lateral recess decompression, intervertebral space dissection, and cage placement. A sterolithography (STL) file was imported to this model to simulate the instrumentation of the body fusion cage and pedicle screws into an appropriate location.

### Digitalized measurement of the ELIF anatomical indices

**Figure 1:**
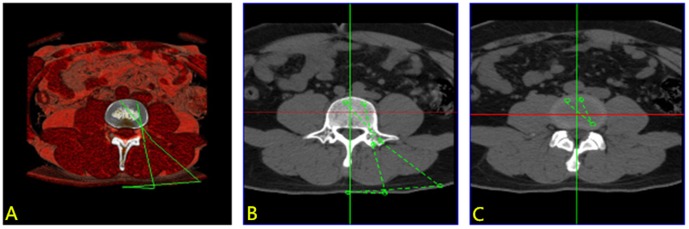
Mimics measurement of anatomical indices on two-dimensional extraforaminal lumbar interbody fusion (ELIF) and TLIF multi-planar rendered images. (A) transverse view of a three-dimensional image; (B) measurement of the distance of the incision to the median line, the depth of the access approach, and the length of the pedicle screw; and (C) measurement of the length of the interbody fusion cage.

Lumbar CT scanning data were obtained from 60 adult outpatients, including 30 males and 30 females aged 18–55 years, none of whom had a known lumbar tumor, inflammation, scoliosis, spondylolisthesis, or congenital spinal malformation. All patients were placed in the supine position, and a 64-detector spiral CT scanner equipped with a SYNGO workstation (SOMATOM Sensation) was used for the lumbar CT scan. The scanning parameters were as follows: tube current  = 250 mA; tube voltage  = 120 kV; scanning slice thickness  = 1.0 mm; and reconstruction slice thickness  = 1.0 mm. The scanning levels included the vertebrae from the T_12_ level to the S_3_ level and the neighboring structures. The DICOM data were imported to Mimics 14.0 equipped with a multifunctional 3D image measurement module to produce axial, sagittal, and coronal two-dimensional (2D) multi-planar rendering (MPR) images. These MPR images were combined with the 3D reconstructed solid images for the measurement of anatomical indices of ELIF at the L_3-4_ and L_4-5_ levels, respectively. These indices included the distance of the incision to the median line, the depth of the access approach, the angle and length of the screw placement, and the length of the instrumented cage (Figure 1). These indices can be obtained by measurement system in Mimics 14.0.

### Mock ELIF on cadaver

Our use of the cadaveric specimens complied with the institutional and national regulations. Two fresh lumbar cadaveric specimens from male donors were provided by the Department of Anatomy, Shanghai Medical College, Fudan University. A longitudinal right paramedian incision (3–5 cm) was made parallel to L_4-5_ and 8–9 cm distant to the median line. The subcutaneous tissue and thoracolumbar fascia were sequentially incised and the intermuscular space was bluntly dissected. The transverse process of the L_5_ vertebra was palpable though an approximate 45° angle. The L_4-5_ facet joint was dissected along the transverse process toward the median line. The vertebral body was exposed along the convergence of the superior process and the transverse process base and slightly toward the ventral side. Upon palpation of the infrapedicular notch, the vertebral body was further dissected toward the ventral side to expose the L_4-5_ disc. The superior part of the intervertebral space was slightly dissected along the orientation of the nerve root to expose the L_4_ nerve root. A Φ2.0-mm orthopedic grinder or boning knife was used to resect ¾ of the superior articular process to fully expose the entire intervertebral foramen, nerve root orientation and exit, and lateral spinal dural sac. The nerve root was well preserved and the intervertebral disc was dissected through the dilated foramen. Following the dissection of the intervertebral space, the interbody fusion cage was instrumented. The convergence of the L_4-5_ superior and transverse processes was drilled and threaded for the placement of the pedicle screws. In the placement of the pedicle screw, the inner wall of the pedicle was explored using a nerve hook, while the nerve root was retracted and well preserved. The connect rod was placed and the screws were tightened. A repeated exploration was performed to ensure good preservation of the nerve root and stability of the internal fixation system prior to ELIF completion.

### Statistical analysis

All data were processed using the SPSS statistical software package (version 20.0; SPSS Inc., Chicago, IL, USA) and expressed as mean ± SD. The means were compared using one-way analysis of variance and the independent samples were compared using Student's *t* test. *P* values<0.05 were considered statistically significantly.

## Results

**Figure 2:**
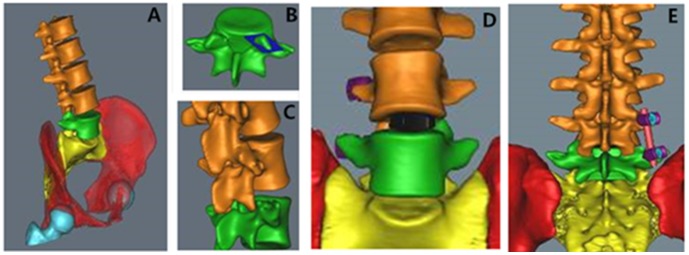
Mimics simulation of extraforaminal lumbar interbody fusion. (A) Three-dimensional reconstruction of the spinal column; (B) extraction of the L_5_ vertebral body and resection of the superior articular process; (C) exposure of the intervertebral space following resection of the superior articular process; (D) placement of the cage through the intervertebral foramen; (E) implantation of the pedicle screws.

The use of the Mimics system enabled 3D solid simulation and vivid preoperative planning for ELIF (Figure 2). The removal of the superior articular process allowed for decompression of the spinal lateral recess and dilation of the foramen, which facilitated further dissection of the intervertebral space and cage placement.

Use of the Mimics also allowed a whole-3D crossover measurement of the target point, line, and plane on a 3D object, which helped the determination of the spatial location and angle. Tables 1 and 2 show the ELIF anatomical measures on the axial, sagittal, and coronal planes in combination with the reconstructed 3D solid model images. The ELIF incision was located approximately 8–9 cm lateral to the median line; the access approach was roughly 8–9 cm; the pedicle screw was 1 cm longer than that used in TLIF; the angle of the screw placement was more inclined, at approximately 40°; and the cage length was approximately 30 mm. Compared to TLIF, ELIF had a more lateral incision that avoided the inferior articular process and allowed for direct access to the hypertrophic, compressed superior articular process through a lateroposterior approach, and the pedicle screw and cage were implanted in a more inclined angle and with a significantly greater length (*P*<0.01).

**Table 1:** L_3-4_ ELIF and TLIF anatomical measures.

		Distance of incision to the central line (mm)	Depth of access approach (mm)	Cage length (mm)	Pedicle screw length (mm)		Pedicle screw inclination (°)	
					L_3_	L_4_	L_3_	L_4_
ETLIF	M	87.0±4.1	85.1±7.4	32.0±1.9	55.2±1.5	55.8±3.0	40.4±2.1	39.1±3.5
	F	83.3±4.0	81.7±6.8	29.8±1.8	51.6±3.5	52.2±2.4	37.7±1.3	37.6±3.2
TLIF	M	38.5±4.4	57.9±4.5	28.4±2.0	47.9±2.1	48.8±3.4	13.8±4.2	11.5±3.6
	F	35.3±4.2	54.3±5.2	26.5±2.1	45.1±4.1	46.0±3.1	12.1±1.6	10.2±3.3

**Table 2:** L_4-5_ ELIF and TLIF anatomical measures.

		Distance of incision to the central line (mm)	Depth of access approach (mm)	Cage length (mm)	Pedicle screw length (mm)		Pedicle screw inclination (°)	
					L_4_	L_5_	L_4_	L_5_
ETLIF	M	87.6±3.8	86.9±4.5	32.2±2.2	55.8±3.0	57.2±1.3	39.1±3.5	39.1±3.5
	F	81.3±3.4	83.0±4.2	30.3±2.0	52.2±2.4	54.4±2.2	37.6±3.2	37.6±3.2
TLIF	M	37.0±4.1	56.4±5.2	28.5±2.2	48.8±3.4	51.3±5.1	11.5±3.6	11.5±3.6
	F	32.9±2.7	54.0±4.9	26.8±2.1	46.0±3.1	48.7±1.2	10.2±3.3	10.2±3.3

**Figure 3:**
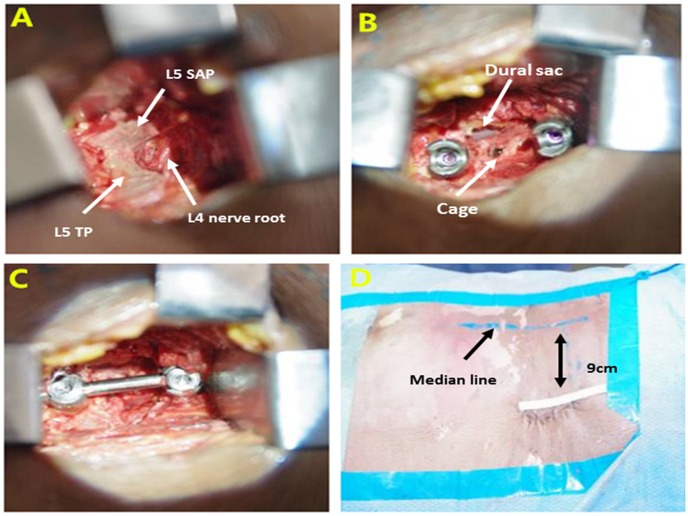
Mock L_4-5_ extraforaminal lumbar interbody fusion on cadaveric specimen. (A) palpation of the L_5_ transverse process and exposure of the superior articular process toward the medial side with preservation of the L_4_ nerve root; (B) resection of the superior articular process, dissection of the intervertebral space, and implantation of the cage and pedicle screws; (C) assembly and fixation of the connect rod; and (D) closure of the incision.

It was feasible to achieve a digitally designed surgical approach, procedure, and decompression on the cadaveric specimens with good consistency with the preoperative planning (Figure 3). Digitalized operative planning showed good accuracy, safety, and stability. The present digitalization technology could not accurately reconstruct images of the minute vascular vessels and nerves, and the mock cadaveric operation was not identical to the actual operation. For example, it was highly risky to access the para- and intraspinal venous plexuses.

## Discussion

Digital medicine is a newly emerging technique which incorporates information technology into clinical medicine. This technique uses a laser scanner, CT, magnetic resonance (MR), and other digital equipment to acquire human body tomographic data. After further integrating these data, spinal digitalized 3D reconstruction can demonstrate the morphology of the spinal column from multiple angles and directions, which allows a clear and full visualization of the disease in whole and in detail. Furthermore, this technique can be used for the selection of surgical approach, the assessment of surgical risk and outcome, and preoperative planning on the digitalized model, for instance, simulating implantation of the cage [8]–[9].

In this study, we used Mimics 14.0 software, which is highly integrated and user-friendly for 3D image production and editing. The Mimics surgical simulation module is a surgical simulation platform for the data analysis of the human regional anatomy and the planning of the skin incision, dissection, and implantation. Compared to conventional gross anatomy measurements and solid operative presentations, 3D reconstruction technology can accurately reproduce the anatomical features of the target structure. The digitalized anatomical model can allow for a 3D dynamic and visualized manipulation in the computer system, which saves materials and time and improves the accuracy of the examination result. In this study, we used multiple digital techniques to simulate key ELIF procedures such as resection of the superior articular process, dissection of the intervertebral space, placement of the cage and pedicle screws, and acquisition of the relevant anatomical measures. All of these results were further validated by the results of the mock operation performed on the cadaveric specimens.

Harms et al. [10] reported a modified TLIF in 1982, which had a more lateral access approach and allowed for lumbar interbody fusion from the posterolateral foramen and the preservation of posterior tension structures such as the supraspinous and interspinous ligaments and laminal attachment of the sacrospinous muscle. Therefore, this modified TLIF has a minimal effect on the mechanical load distribution of the spinal column. Additionally, this modified procedure avoids excessive retraction of the dural sac and nerve roots and reduces the risk of intraspinal venous plexus bleeding and nerve root injury. As a major development in the concept of minimally invasive spinal surgery, Holly et al. [11] innovated a minimally invasive TLIF based on conventional TLIF, which had an access approach through the intermuscular space between the multifidus muscle and the longissimus dorsi muscle. TLIF procedures could be completed inside the catheter and minimize operative injuries. Compared to PLIF, TLIF preserves the spinous process and lamina that are not causative of neurological symptoms, while only the symptomatic lateral spinal canal is specifically decompressed with a great emphasis on the maximized preservation of the lumbar muscles and bony structure [12]. As the inferior articular process overlies the dorsal side of the superior articular process, TLIF requires the preceding resection of the inferior and superior articular process for nerve root decompression and intervertebral space dissection. However, the inferior articular process is not the etiological cause in most cases, but it is sacrificed for the establishment of the access approach and the surgical field [13]. As inspired by the development of Joimax foraminal endoscopy, we designed a more lateral approach than TLIF, namely ELIF. This approach passes through the intermuscular space between the multifidus muscle and the longissimus dorsi muscle and avoids the inferior articular process. It also allows for the direct exposure and resection of the superior articular process that causes neurological symptoms and enables the decompression of the lateral spinal canal and interbody fusion in the safety angle. ELIF is even more minimally invasive since it preserves the posterior tension structures including the inferior articular process, lamina, and attaching ligaments and muscles. The superior articular process is partially resected, and the residual portion can still articulate with and support the inferior articular process. The more inclined approach in ELIF results in a more inclined (45° or even coronal) cage implantation compared to that in TLIF. Therefore, a lengthened cage needs to be placed since it can increase the bone grafting bed area, prevent cage subsidence, and improve the fusion rate. Moreover, the use of more inclined and longer pedicle screws can penetrate the three columns of the vertebral bodies, which enhances the mechanical strength and results in good stability with unilateral fixation [14], [15]. Our digital anatomical measurement results confirmed that ELIF had a more inclined placement of pedicle screws using longer pedicle screws and a longer cage compared to TLIF. Furthermore, ELIF can make direct decompression in lateral and even part of central canal which is totally different in ALIF and X/DLIF. But ELIF also has some potential shortcomings compared to TLIF. The more lateral incision of ELIF make the approach much deeper than TLIF, so this places a greater burden on lighting in operation site and surgical skills. And we can't make reduction because the inferior facet joint is left intact, so it's difficult to treat spondylolisthesis with ELIF.

We used the simulated ELIF to determine the anatomical indices in the mock ELIF on the cadaveric specimens. The incision was made 9 cm distal to the median line, and the approach passes the intermuscular space and reaches the transverse process. Further dissection along the transverse process toward the mediosuperior side was made to fully expose the superior articular process and reach the intervertebral foramen. The exit nerve is located on the ventral side of the inter-transverse-process ligament as a leg of the safety triangle, and the ligament is appropriately dissected to identify the nerve root. It is essential to locate the nerve root in a retrograde manner and preserve the nerve root during placement of the superior pedicle screw [16], [17]. Resection of the superior articular process can fully expose the intervertebral foramen, which is located distal to the dural sac and the inferior nerve root. The exit nerve root should be properly preserved during intervertebral space decompression and cage placement. In the placement of the pedicle screw, the inner wall of the pedicle is explored using a nerve hook and adjustment of the inclining angle (approximately 40°) while avoiding the entrance to the spinal canal.

According to the computer-based simulation and cadaveric study, it is feasible to perform ELIF. In conclusion, ELIF is a more inclined approach than TLIF and the lateral resection of the symptomatic superior articular process allows for better disc dissection and cage placement. Further research including biomechanical study is needed to prove ELIF has a superior ability to preserve the posterior tension bands of the spinal column, with similar effects on spinal decompression, fixation, and fusion, and it can enhance post-fusion spinal stability and expedites postoperative recovery. However, this approach also has some disadvantages such as the intervertebral foramen area has abundant venous plexuses and is at high risk of bleeding and nerve injury, it is also difficult to decompress centrally and need a steep learning curve to perform ELIF.
